# Super-enhancer-associated transcription factors collaboratively regulate trophoblast-active gene expression programs in human trophoblast stem cells

**DOI:** 10.1093/nar/gkad215

**Published:** 2023-03-23

**Authors:** Mijeong Kim, Enoch Appiah Adu-Gyamfi, Jonghwan Kim, Bum-Kyu Lee

**Affiliations:** Department of Molecular Biosciences, Center for Systems and Synthetic Biology, The University of Texas at Austin, Austin, TX 78712, USA; Department of Biomedical Sciences, Cancer Research Center, University at Albany, State University of New York, Rensselaer, NY 12144, USA; Department of Molecular Biosciences, Center for Systems and Synthetic Biology, The University of Texas at Austin, Austin, TX 78712, USA; Department of Biomedical Sciences, Cancer Research Center, University at Albany, State University of New York, Rensselaer, NY 12144, USA

## Abstract

The placenta is an essential organ that supports the growth and development of the fetus during pregnancy. However, cell type-specific enhancers and transcription factors (TFs), and the mechanisms underlying the maintenance and differentiation of trophoblast stem cell (TSC) populations in the human placenta remain elusive. Here, using human TSCs as a model system, we identify 31,362 enhancers that are enriched with the motifs of previously reported TSC-pivotal TFs, including TEAD4, GATA2/3 and TFAP2C. Subsequently, we identify 580 super-enhancers (SEs) and 549 SE-associated genes. These genes are robustly expressed in the human placenta and include numerous TFs, implying that SE-associated TFs (SE-TFs) may play crucial roles in placental development. Additionally, we identify the global binding sites of five TSC-pivotal SE-TFs (FOS, GATA2, MAFK, TEAD4 and TFAP2C), revealing that they preferentially co-occupy enhancers, regulate each other and form a trophoblast-active gene regulatory network. Loss-of-function studies unveil that the five TFs promote self-renewal of TSCs by activating proliferation-associated genes while repressing developmental genes. We further reveal that the five TFs exert conserved and unique functions on placental development between humans and mice. Our study provides important insights into the roles of human TSC-pivotal TFs in regulating placenta-specific gene expression programs.

## INTRODUCTION

The placenta is a short-lived but vital organ that sustains the growth of the fetus during pregnancy. It originates from the outer part of the blastocyst, which is called the trophectoderm, and plays versatile roles, including hormone production, oxygen and nutrient supply to the fetus, fetal waste removal and fetal protection (1). The human placenta consists of three major trophoblast lineages: cytotrophoblasts (CTs) which are multipotent progenitor cells; syncytiotrophoblasts (STs) which are responsible for hormone production and feto-maternal exchanges of gases and nutrients; and extravillous trophoblasts (EVTs) that can invade the maternal decidua and remodel its blood vessels to establish the uteroplacental circulation (2). Abnormal trophoblast differentiation is closely linked to placenta-related disorders, including stillbirth, miscarriage, intrauterine fetal growth restriction (IUGR) and pre-eclampsia (PE) (3). Despite the significant roles of trophoblasts in establishing the feto-maternal interface, the molecular mechanisms underlying the maintenance and differentiation of trophoblast progenitors and their relevance to placental pathologies remain poorly understood.

Recently, human trophoblast stem cells (TSCs) were derived from the blastocyst and the first trimester placenta (4). TSCs are considered *in vitro* counterparts of CTs, since they can self-renew and exhibit multipotency that gives rise to STs and EVTs. As human TSCs can faithfully recapitulate the *in vivo* differentiation of CTs toward STs and EVTs, they are considered reliable *in vitro* models for studying human placental development and placenta-associated pregnancy complications.

Gene expression programs are precisely controlled by orchestrated actions of cell type-specific transcription factors (TFs) and enhancers that function as hubs to integrate chromatin state and TF binding (5). Combinatorial usage of enhancers and various tissue-specific TFs in complex transcriptional gene regulatory networks confers divergent gene expression programs which underpin distinct cellular functions, identities and phenotypic outcomes (6). In addition, recent studies proposed that ‘super-enhancers’ (SEs)—dense clusters of enhancers—are associated with key cell type-specific TFs and control unique cellular identities and diseases (7,8). Thus, identifying global enhancers, SEs and SE-associated TFs (SE-TFs) as well as their binding sites in human TSCs is a prerequisite to understanding the mechanisms underlying the maintenance and multipotency of TSCs as well as human placental development.

Mouse genetic studies revealed that multiple TFs such as Cdx2 (9), Tead4 (10), Gata2/3 (11), Tfap2c (12) and Elf5 (13) play crucial roles in placental development. Additionally, genome-wide enhancers, putative TSC-pivotal TFs and the global binding sites of some of the TFs were identified in mouse TSCs (14–16). In contrast, only a limited number of human TSC-pivotal TFs and *cis*-regulatory elements have been reported. In addition, their functions in human TSCs have not been thoroughly addressed. Importantly, it is largely unknown to what extent key human TSC-active TFs share their targets and exert conserved or distinct functions between humans and mice. Even though human and mouse placentas play equivalent roles and have numerous functionally conserved genes, there are some discrepancies in their morphology, cellular organization and gene expression patterns (17–19). Considering these apparent differences, it is necessary to identify human TSC-specific enhancers, TFs and their targets in order to comprehensively understand the regulatory mechanisms underlying human placental development.

Here we investigated the enhancer landscape of human TSCs using chromatin immunoprecipitation followed by high-throughput sequencing (ChIP-seq) of the enhancer-associated protein EP300 and subsequently defined SEs and SE-associated genes. SE-associated genes include many TFs that are highly expressed in the human placenta. To gain insights into the molecular mechanism by which TSC-pivotal TFs modulate placenta-specific gene expression, we also mapped the global binding sites of five TSC-pivotal SE-TFs in human TSCs, revealing that they collaboratively regulate placenta-specific gene expression programs.

## MATERIALS AND METHODS

### Cell culture

Human TSCs were obtained from Dr. Takahiro Arima and cultured as previously described (4). Briefly, the human TSCs were seeded on 5 μg/ml Collagen IV (Corning)-coated plates containing TSC culture medium [Dulbecco’s modified Eagle’s medium (DMEM)/F12 (Gibco)]. The medium was supplemented with 1% insulin-transferrin-selenium-ethanolamine (ITS-X; Gibco), 0.3% bovine serum albumin (BSA; Sigma-Aldrich), 0.2% fetal bovine serum (FBS; GeminiBio), 0.1 mM β-mercaptoethanol (Sigma-Aldrich), 0.5% penicillin–streptomycin (Gibco), 0.5 μM A83-01 (Wako Pure Chemical Corporation), 0.5 μM CHIR99021 (Selleck Chemicals), 0.5 μM SB431542 (Stemcell Technologies), 5 μM Y27632 (ROCK inhibitor, Selleck Chemicals), 0.8 mM valproic acid (VPA; Wako Pure Chemical Corporation), 50 ng/ml epidermal growth factor (EGF; PeproTech) and 1.5 μg/ml l-ascorbic acid (Sigma-Aldrich). When the cells reached 70–80% confluency, they were trypsinized with TrypLE (Gibco) for 8 min at 37°C and the detached cells were seeded on Collagen IV-coated dishes at a 1:3 split ratio. TSCs were cultured in a 37°C and 5% CO_2_ incubator. The medium was replaced every day with a fresh medium.

### Virus preparation and shRNA-mediated knockdown (KD) of TFs

Short hairpin RNAs (shRNAs) against FOS, GATA2, MAFK, TEAD4, TFAP2C, NCOR2, RCOR1 and ZNF362 were purchased from Sigma-Aldrich (Supplementary Table S1). A total of 7.3 × 10^6^ HEK293T cells were transfected with 2.5 μg of the pLKO construct with a specific shRNA, 1.7 μg of Δ8.9 and 0.8 μg of VSVG using 15 μl of a GenJet transfection reagent (SignaGen) to generate lentiviruses expressing a specific shRNA. After 24 h, the medium was replaced with the TSC medium. After cultivating at 37°C for 24 h, the viral supernatant was collected, filtered with a syringe filter (0.45 μm) and used to infect TSCs. To knock down individual TFs, 2.5 × 10^5^ TSCs were infected with pLKO-shRNA viral particles in a well of a 12-well plate. Infected cells were incubated overnight, and the medium was replaced with fresh TSC medium supplemented with puromycin to select out the infected cells. To determine the KD efficiency of each TF and profile global gene expression, we harvested the cells after 3 days of selection to get complete KD of an individual TF.

### Cell proliferation assay

TSCs were infected with pLKO-shRNA viral particles for 24 h, and then cultured in TSC medium supplemented with puromycin for 2 days. The infected cells were seeded at a density of 2.1 × 10^4^ cells/ml in 96-well plates. Proliferation was measured using the cell counting kit-8 (Dojindo, CK04) following the manufacturer's instructions at four time points beginning on the day of seeding. Briefly, the medium was aspirated from each well and replaced with 100 μl of TSC medium with 10 μl of CCK-8 reagent. The plates were incubated at 37°C for 2 h and their absorbance was measured with the Tecan M1000 Plate Reader at 450 nm.

### Apoptosis assay

TSCs were infected with pLKO-shRNA viral particles for 24 h, and then cultured in TSC medium supplemented with puromycin for 3 days. Apoptotic cell populations were measured using the NucView Dual Apoptosis Assay Kit for Live Cells (Biotium, 30067). The infected cells were dissociated into single cells and washed with phosphate-buffered saline (PBS). The cells were resuspended in 200 μl of 1× Annexin V binding buffer with 5 μl of Sulforhodamine 101–Annexin V (Texas Red) stock solution and incubated for 30 min at room temperature, protected from light. Cells were washed once with 1× Annexin V binding buffer and filtered using a cell strainer (70 μm filter, Falcon, 352235). Flow cytometry was performed on a BD LSRFortessa SORP Flow Cytometer (BD Biosciences) and the data were analyzed with FlowJo (v9, Treestar).

### Reverse transcription–quantitative polymerase chain reaction (RT–qPCR)

Complementary DNA (cDNAs) were generated from 500 ng of total RNA using qScript (Quanta) and then diluted 20 times with DNase-free water. A 2 μl aliquot of cDNA was used for one RT–qPCR using a PerfeCTa SYBR Green FastMix (Quanta). PCR primers were designed to amplify the junction between two exons using a web-based primer design program, Primer3 (http://bioinfo.ut.ee/primer3/). All primer sequences are listed in Supplementary Table S1. Cycle threshold (Ct) values of samples and controls were normalized against glyceraldehyde-3-phosphate dehydrogenase (GAPDH) as a loading control, and then relative gene expression was calculated as fold enrichment using the 2^–ΔΔCT^ method. The relative expression of a gene was calculated from an average of three replicates.

### RNA sequencing (RNA-seq)

Global gene expression was profiled using RNA-seq in human TSCs. Total RNAs were isolated using the RNeasy Plus Mini Kit (Qiagen, 74136). RNA-seq libraries were generated with 500 ng of total RNA using the NEB Next Ultra II RNA Library prep kit (NEB, E7770) following the manufacturer's protocol. Briefly, mRNAs were enriched from total RNAs with the Magnetic mRNA Isolation Kit [oligo(dT) beads] (NEB, E7490), and then cDNAs were synthesized using random primers and purified by NEBNext Sample Purification Beads (NEB, E7767). The ends of purified double-stranded cDNAs were repaired, ligated with a barcoded adaptor and amplified with PCR. RNA-seq libraries were sequenced using an Illumina NovaSeq 6000 platform. Paired-end reads were aligned to human transcript (hg38) using the Salmon mapper (v1.4.0) (20). Expression levels of each gene were calculated using the R library tximport (v1.18.0) (21) as transcripts per million (TPM). The read counts were quantified and normalized by the median of ratio method using the R package DESeq2 (v1.30.1) (22), and differentially expressed genes (DEGs) were obtained with the selection criteria of *P*-adjusted value <0.01 and fold change (FC) >| 1 |.

### ChIP-seq

ChIP experiments were conducted as previously described (23). Briefly, TSCs were fixed with 1% formaldehyde for 7 min at room temperature, and then glycine (final 125 mM) was added to quench formaldehyde (5 min). The fixed cells were sonicated using a Bioruptor (Diagenode) with a setting of 30 s on and 1 min off for 10 min (total of three times), and the sheared chromatins that have an average of 250 bp DNA fragments were utilized for immunoprecipitation using 10 μg of a native antibody. The antibodies used include EP300 (Santa Cruz, sc-585), FOS (Santa Cruz, sc-7202X), GATA2 (Santa Cruz, sc-9008X), MAFK (Santa Cruz, sc-477X), TEAD4 (Abcam, ab58310), TFAP2C (Santa Cruz, sc-8977) and H3K27ac (Active Motif, 39133). Enriched ChIP materials were used to generate next-generation sequencing libraries using an NEB ChIP-seq library preparation kit (NEB, E7370L). ChIP-seq libraries were sequenced using an Illumina HiSeq 2500 machine.

### ChIP-seq data processing

Single reads (of 50 bp) from ChIP-seq were mapped onto the human genome assembly (hg38) using the Bowtie2 mapper (24), and low-quality reads were filtered out (MapQ <10). Mapped reads were used for peak calling with the model-based analysis for ChIP-seq (MACS3) peak caller (25) with a default setting except for H3K27ac peak calling, where we used –max-gap 1000. Further score filtering was applied to remove weak peaks by visual inspection. The human genome hg38 repeat-mask file was downloaded from the University of California Santa Cruz (UCSC) table genome browser (https://genome.ucsc.edu/cgi-bin/hgTables). Peaks found in simple redundant regions of the genome were further filtered out.

### Identification of super enhancers

SEs were defined using the ROSE program downloaded from the website of the Young lab (http://younglab.wi.mit.edu/super_enhancer_code.html). We first identified the binding sites of EP300 using the MACS3 peak caller, and then the defined peaks were transformed into general feature format (GFF) files to meet the criteria of input files of the ROSE program. We ran the ROSE with options of a stitching distance (12.5 kb) and transcription start site (TSS) exclusion zone size (2.5 kb).

### Profiling ChIP-seq signals in a given region

A region of ±3 kb from the center of a peak was binned (100 bp), and all reads from ChIP-seq were mapped into each bin. The score in each bin was calculated by summing up the number of reads assigned into a bin. The score was also normalized with a sequencing depth. Average bin scores across the regions were plotted to generate averaged read density across the regions.

### Mapping peaks to gene features

To identify the target genes of a TF, peaks were mapped to the region surrounding ±20 kb from the TSS of all RefSeq genes in the RefFat file downloaded from the UCSC genome browser. To assign a binding site to one genomic feature, we used the following hierarchy: promoter > intron > exon > intergenic regions. A promoter was a region within ±2 kb from the TSS. Binding sites except promoters, introns or exons were considered as intergenic regions.

### Motif analysis

Enriched motifs of each TF were searched using findMotifsGenome.pl in the Homer motif analysis tool (http://homer.ucsd.edu/homer/motif/ HOMER). Central motif enrichment was further examined by annotatePeaks.pl with -hist option in Homer.

### Overlap analysis

Overlapping binding sites among ChIP-seq data were identified using a moving window across the human genome. When the centers of the peaks from different ChIP-seq data were positioned within a 500 bp window, we considered them as overlapping peaks.

### Gene Ontology analysis

To identify enriched Gene Ontology (GO) terms, the Genomic Regions Enrichment of Annotation Tool (GREAT) was used for ChIP-seq data (26). To avoid the saturation of the gene-based hypergeometric test, we used the top 5,000 strong binding sites of a TF. GREAT (26) and Metascape (27) were also used to identify enriched biological pathways and protein complexes for common and unique targets of TFs.

### Correlation analyses

A pair-wise Pearson correlation coefficient between the target genes of two TFs was calculated for each pair of TFs. Clustering analysis and visualization of the data were done with Cluster 3.0 (28) and Java Treeview (29), respectively.

### Network construction and visualization

To assign a binding site to a gene, TF-binding sites were mapped to the region surrounding 20 kb upstream and downstream of the TSS as well as the gene body of all RefSeq genes from the RefFlat file obtained from the UCSC genome browser. When a binding site was not found within ±20 kb including the gene body, the nearest gene was assigned as the target of the binding site. TFs and target pairs were visualized using Cytoscape (http://www.cytoscape.org).

### Data deposited or used for analyses

All sequencing data we generated were uploaded onto the Gene Expression Omnibus (GEO) with accession number GSE208539. Processed human TSC, ST and EVT RNA-seq data were obtained from the published paper of Okae *et al.* (4). For PE analysis, RNA-seq data were downloaded from the GEO with accession number GSE114691.

## RESULTS

### Enhancer landscape of human TSCs

**Figure 1.**
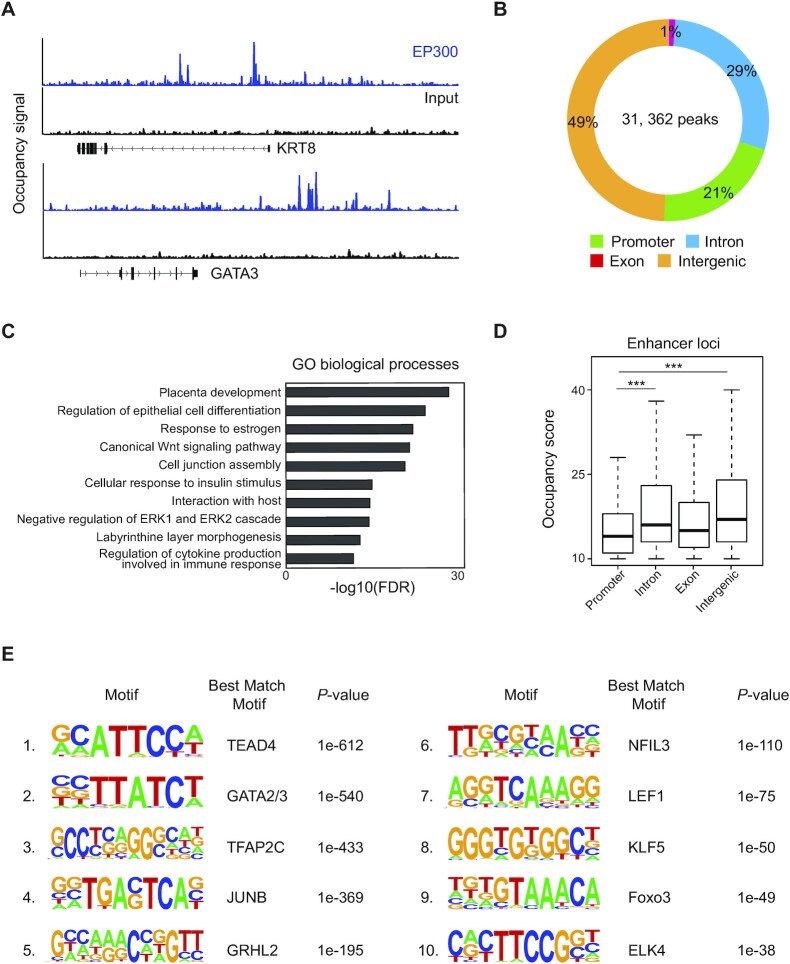
ChIP-seq of EP300 in human TSCs revealed global enhancers enriched with the motif of TSC-pivotal TFs. (**A**) EP300 ChIP-seq (blue) and input (black) signal tracks near TSC marker genes. (**B**) A pie chart displaying the distribution of EP300-binding sites across the genome. Promoters are within 2 kb from the TSSs, and 'intergenic' is a region excluding a promoter, intron and exon. (**C**) Enriched GO terms of biological processes in the EP300-binding sites. (**D**) A boxplot presenting the distribution of EP300 occupancy scores across different genomic loci. *P*-value was calculated using the Wilcoxon rank sum test. ***P-value <0.0001. (**E**) Top 10 enriched motifs in the EP300-binding sites.

To investigate the global landscape of enhancers and understand enhancer usages in human TSC, we performed ChIP-seq of an enhancer-associated protein EP300. As shown in Figure 1A, strong occupancy signals of EP300 were observed around TSC marker genes (KRT8 and GATA3), suggesting TSC-specific functions of the enhancers. We identified a total of 31,362 binding sites of EP300. Like other tissue type-specific enhancers (30), the vast majority of the EP300-binding sites (49% of intergenic loci and 29% of introns) were found distal from the TSSs, while 21% of the sites were in promoters (Figure 1B). As enhancers govern cellular functions and identity by establishing cell type-specific gene expression programs, we performed GO analysis to see whether the enhancers in TSCs regulate placenta-specific gene expression. The targets of EP300 were significantly enriched for placenta development-associated terms, including placenta development, response to estrogen and labyrinthine layer morphogenesis (Figure 1C). Notably, compared with promoters, distal enhancers showed higher occupancy scores of EP300, implying that the distal enhancers play key roles in TSCs (Figure 1D). Consistent with this notion, the distal enhancers presented stronger enrichment in a GO term of placenta development than the promoters (Supplementary Figure S1A).

Since multiple TFs can co-occupy an enhancer in close proximity and form an enhanceosome to stimulate gene activation (31), we explored the sequences in the enhancers to identify the enriched motifs of TFs. The motifs of previously reported trophoblast-pivotal TFs such as TEAD4 (32), GATA2/3 (33–35) and TFAP2C (36,37) were top ranked (Figure 1E). TFs whose motifs were enriched in TSC enhancers were more robustly expressed in CTs and TSCs than in other cell types in the human placenta (Supplementary Figure S1B). In agreement with the strong EP300 occupancy, the distal enhancers showed the strongest enrichment of TSC-active TF motifs (Supplementary Figure S1C). In sum, we successfully identify genome-wide enhancers of human TSCs, revealing that they are enriched with multiple TSC-pivotal TF motifs and involved in human placental development.

### SEs regulate human TSC-pivotal TFs

**Figure 2.**
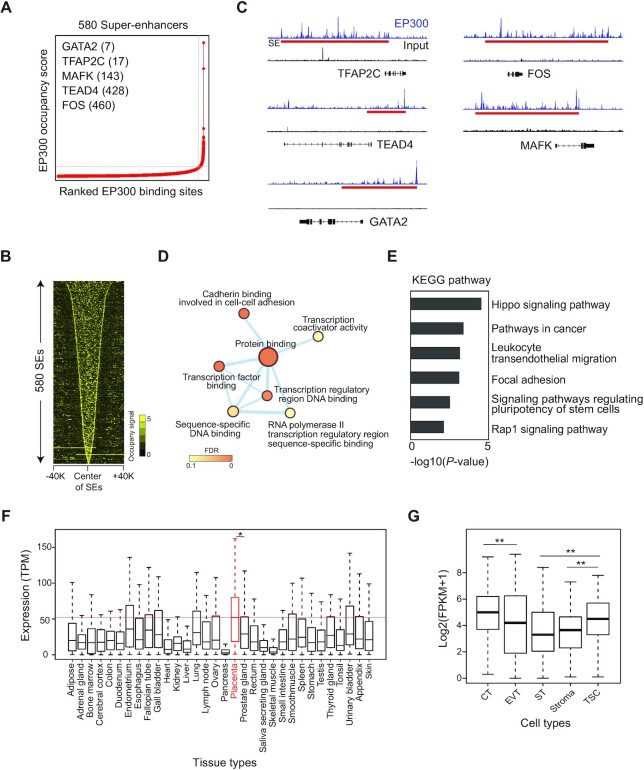
SEs control trophoblast-active genes in human TSCs. (**A**) A line graph showing the number of SEs defined by the ranked EP300 binding signal. (**B**) A heatmap showing the EP300 occupancy signal around the center of SEs. (**C**) EP300 ChIP-seq (blue) and input (black) signal tracks near TFs. A red rectangular box indicates an SE. (**D**) A network map displaying GO terms enriched in molecular functions in SE-associated genes. Node color indicates the false discovery rate (FDR), and the thickness of the line presents the degree of overlapped genes between two terms. (**E**) KEGG pathways enriched in SE-associated genes. (**F** and **G**) Boxplots showing the distribution of expression of SE-TFs in various human tissues (F) and cell types (G). The red line indicates a median expression value among the genes in the placenta tissue. *P*-value was calculated using the Wilcoxon rank sum test. *P-value <0.01; **P-value <0.001; ns not significant.

Since SEs determine cell type-specific gene expression (7), we sought to identify key TSC-active TFs by mapping SEs. With the ROSE program (38), we identified 580 SEs and 549 SE-associated genes, among which 76 genes were TFs (Figure 2A; Supplementary Table S2). As expected, SEs were broad and had higher EP300 occupancy signals than typical enhancers (TEs) (Figure 2B; Supplementary Figure S2A). SE-associated genes include previously reported TSC-pivotal TFs such as TEAD4, GATA2/3, TFAP2C and MSX2 (39) (Figure 2C; Supplementary Table S2), while numerous TFs whose functions have not been reported in placental biology were also identified. To validate our SEs, we also performed ChIP-seq of H3K27ac, which is an active enhancer mark (40), and identified SEs (Supplementary Table S3). As shown in Supplementary Figure S2B, 68% of SEs defined by EP300-binding sites overlapped with SEs defined by an H3K27ac. Previously reported trophoblast-active TFs including TEAD4, GATA2/3, TFAP2C, FOS and MAFK were associated with the SEs defined by both EP300 and H3K27ac ChIP-seq data. Additionally, we knocked down three novel TF candidates (NCOR2, RCOR1 and ZNF362) that are associated with the SEs. Depletion of these TFs disrupted the homogeneous shape of the TSCs and displayed abnormal and larger cell sizes, and the cells showed decreased levels of TSC-active genes (Supplementary Figure S2C, D) along with reduced proliferation rates (Supplementary Figure S2E). These results suggest that SE-predicted TFs play important roles in the self-renewal of TSCs.

To examine the significance of SE-associated genes in TSCs, we performed GO analysis, revealing that SE-associated genes are highly enriched for placental development and molecular functions of TF binding, suggesting that SE-TFs play important roles in placental development (Figure 2D; Supplementary Figure S2F). In higher eukaryotes, enhancers exert pivotal roles in integrating diverse signaling pathways to stimulate distinct gene expression (41). Interestingly, Kyoto Encyclopedia of Genes and Genomes (KEGG) pathway analysis revealed that SE-associated genes are enriched for Hippo signaling, signaling pathways in cancer and signaling pathways regulating pluripotency of stem cells, implying that they are involved in cell proliferation and differentiation (Figure 2E). Given the fact that SEs control cell type-specific TFs (38), we inspected the expression levels of SE-TFs in multiple human tissues to see whether SE-TFs are highly expressed in the human placenta. As shown in Figure 2F, SE-TFs displayed the highest expression in the placenta among the tissues examined. In particular, SE-TFs are relatively highly expressed in CTs and human TSCs compared with the other trophoblast subtypes and the placental stroma cells (Figure 2G), suggesting that they play roles in the maintenance of stem cells in the placenta. Taken together, these results suggest that TSC-specific SEs play critical roles in placental development by regulating TSC-pivotal TFs.

### TSC-specific TFs collaboratively regulate their target genes

**Figure 3.**
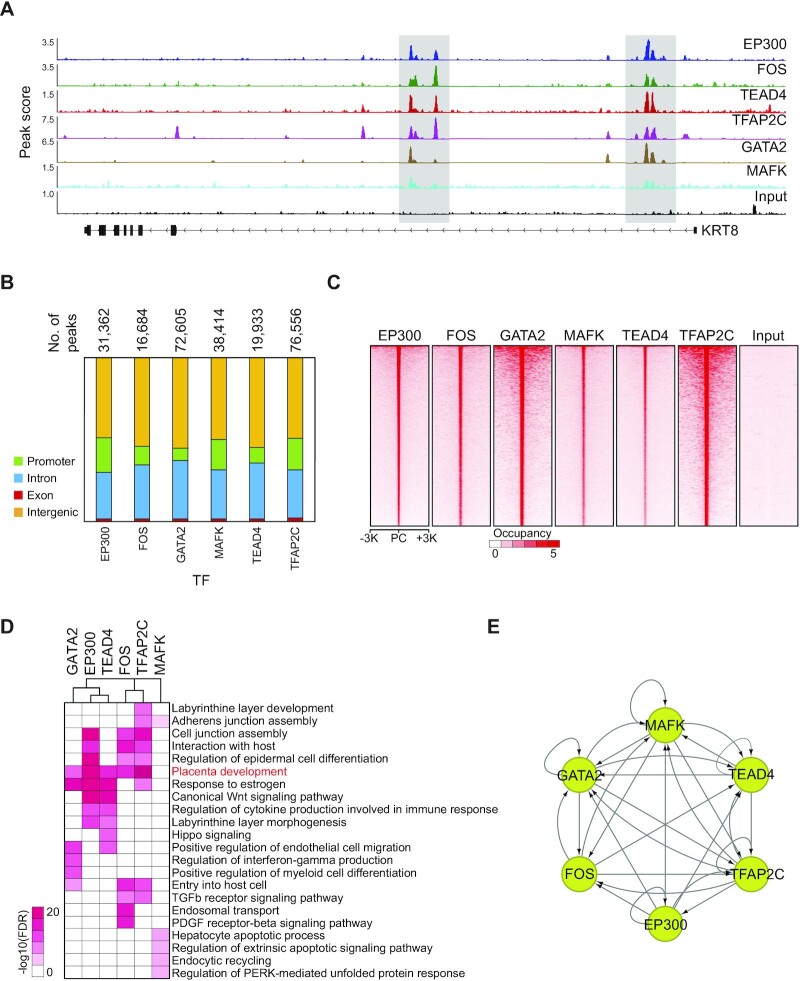
TSC-pivotal TFs collaborate to activate placenta developmental genes. (**A**) ChIP-seq signal tracks of multiple SE-TFs, EP300 and input near trophoblast-specific gene, KRT8. (**B**) Distribution of TF-binding sites on the genome. The total number of binding sites is shown on the bar graph. Each color indicates a different genomic region. (**C**) Heatmaps presenting occupancy signals of various SE-TFs near the center of EP300-binding sites. PC indicates a peak center. (**D**) A heatmap showing enriched GO terms in the binding sites of various TFs. The color bar indicates a negative log-transformed FDR. (**E**) A network map illustrating a transcriptional gene regulatory network among various SE-TFs. An arrow points at the target of a TF.

To interrogate the molecular mechanisms by which TSC-pivotal TFs modulate human placental development, we selected five SE-TFs (FOS, TEAD4, TFAP2C, GATA2 and MAFK) that are robustly expressed in both TSCs and CTs (Figure 2C; Supplementary Figure S2G) and performed ChIP-seq of them in human TSCs. All five TFs exhibited strong occupancy signals at the enhancers of a TSC marker gene, KRT8 (Figure 3A), indicating the decent quality of the ChIP-seq data. To further evaluate the quality of the ChIP-seq data, we conducted motif analyses to see whether the binding sites of a TF are enriched with its canonical motif. The motif corresponding to an individual TF was significantly over-represented in the binding sites of the TF, which is indicative of the good quality of the data (Supplementary Figure S3A). With these ChIP-seq data, we identified tens of thousands of the binding sites and target genes of each TF (Figure 3B; Supplementary Table S4). Intriguingly, these five TFs prefer to co-occupy the distal regulatory loci such as intergenic regions (Figure 3B, C), implying that the TFs collaborate to regulate TSC-active genes by binding together at the distal enhancers of TSCs. In line with this, GO analyses disclosed that the term ‘placenta development’ was strongly enriched in the targets of FOS, GATA2, TEAD4 and TFAP2C (Figure 3D). Although somewhat different GO terms were enriched for an individual TF, we found that these five TFs are prone to bind together (Figure 3C). Previous studies suggested that TFs in embryonic stem cells are involved in an intricate transcriptional gene regulatory network with autoregulation, feed-forward regulation and interconnectivity (42,43). Similarly, we observed all three regulatory modes in the network of the five TFs along with EP300 in TSCs (Figure 3E). All these data suggest that the five SE-TFs make up a complex gene regulatory network and collaboratively regulate the genes implicated in human placental development.

### Co-occupancy of TSC-pivotal TFs on enhancers is crucial for regulating placenta-specific genes

**Figure 4.**
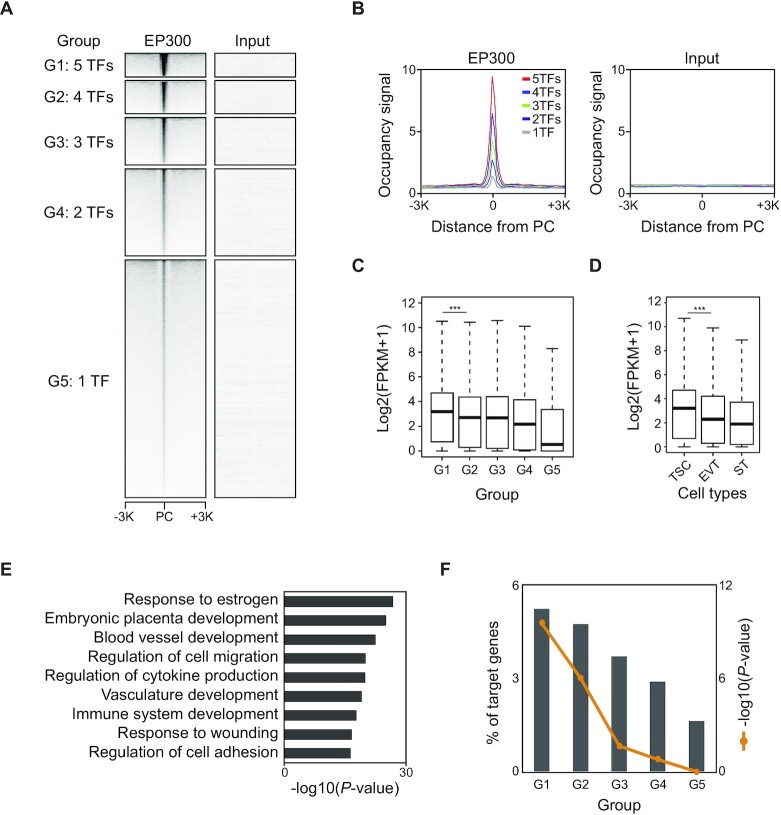
Co-occupancy of TSC-pivotal TFs is critical to the regulation of TSC-specific and PE-deregulated genes. (**A**) Heatmaps presenting EP300 occupancy signals at the center of peaks belonging to each group (G1 to G5) that is classified based on the number of TFs co-bound. PC indicates a peak center. (**B**) Line graphs showing averaged EP300 occupancy (left) and input (right) signals near the center of peaks in the five different groups. (**C** and **D**) Boxplots showing the expression distribution of an individual group-associated gene in TSCs (C) and the G1-associated genes in various trophoblast cell types (D). *P*-value was calculated using the Wilcoxon rank sum test. ***P-value <0.0001. (**E**) Enriched GO terms of biological processes of G1-associated genes. (**F**) Percentage of genes in the five groups that are overlapped with PE-deregulated genes. *P*-values were calculated using a hypergeometric test. PE data were obtained from GSE114691.

Since human TSC-pivotal TFs co-occupy many enhancers of TSCs (Figure 3C), we explored to what extent this co-occupancy influences the binding of an individual TF and the expression of a TF’s target genes among the five TFs. First, we examined the regions bound by multiple TFs and then classified them into five groups (G1 to G5) based on the number of TFs co-bound (Figure 4A; Supplementary Table S5). Next, we examined the EP300 occupancy in each group. As shown in Figure 4A and B, the regions co-bound by the five TFs showed the most robust EP300 occupancy, implying that TSC-pivotal TFs preferentially co-occupy the strong enhancers. This result is consistent with the fact that numerous binding sites of TFs are densely clustered within relatively compact genomic regions, which is a feature of the enhanceosome (31).

To assess whether these five TFs co-occupy the enhancers in a cooperative manner, we investigated the occupancy of TFs along with the number of TFs co-bound. Stronger occupancy of a TF was observed in the regions with a higher number of TFs co-bound (Supplementary Figure S4A), implying that TSC-active TFs strengthen their target binding in a cooperative manner. We then compared gene expression among the groups. G1 targets showed the highest expression, whereas G5 targets had the lowest expression in TSCs. Importantly, the expression of the targets of TFs increased along with the number of TFs co-occupied, suggesting that binding of multiple TSC-pivotal TFs enhanced their target gene expression (Figure 4C). This observation demonstrates that TSC-pivotal TFs act collaboratively to activate a subset of their target genes, whereas they are predominantly inactive or repressive on their targets when functioning alone or with only a few other TFs. This is in line with the notion that efficient transcription requires the combinatorial binding of TFs to enhancers, which confers transcriptional synergy (44).

We also interrogate the biological relevance of co-occupancy of TFs by comparing the co-occupancy with the expression of G1 targets in self-renewing TSCs and differentiated cells, including STs and EVTs. As expected, G1 targets presented higher expression in TSCs and CTs than STs and EVTs (Figure 4D; Supplementary Figure S4B), indicating that they are generally active in self-renewing stem cells in the placenta. Additionally, GO analyses for each group of targets disclosed that G1-associated genes are the most significantly enriched for placenta development and they were placenta-specifically expressed (Figure 4E; Supplementary Figure S4C). As defects in placental development are often linked to pregnancy complications such as PE (45), we examined the overlaps of target genes of each group with PE-deregulated genes. We found that PE-deregulated genes were the most significantly enriched in G1 targets. The percentage of these genes increased along with the number of TFs co-bound (Figure 4F). Collectively, these results suggest that TSC-active SE-TFs co-occupy the enhancers of TSCs and control placental development-related genes, and that aberrant expression of the genes regulated by multiple TSC-active SE-TFs is implicated in the pathogenesis of PE.

### TSC-pivotal TFs directly activate proliferation-promoting genes while repressing developmental genes

**Figure 5.**
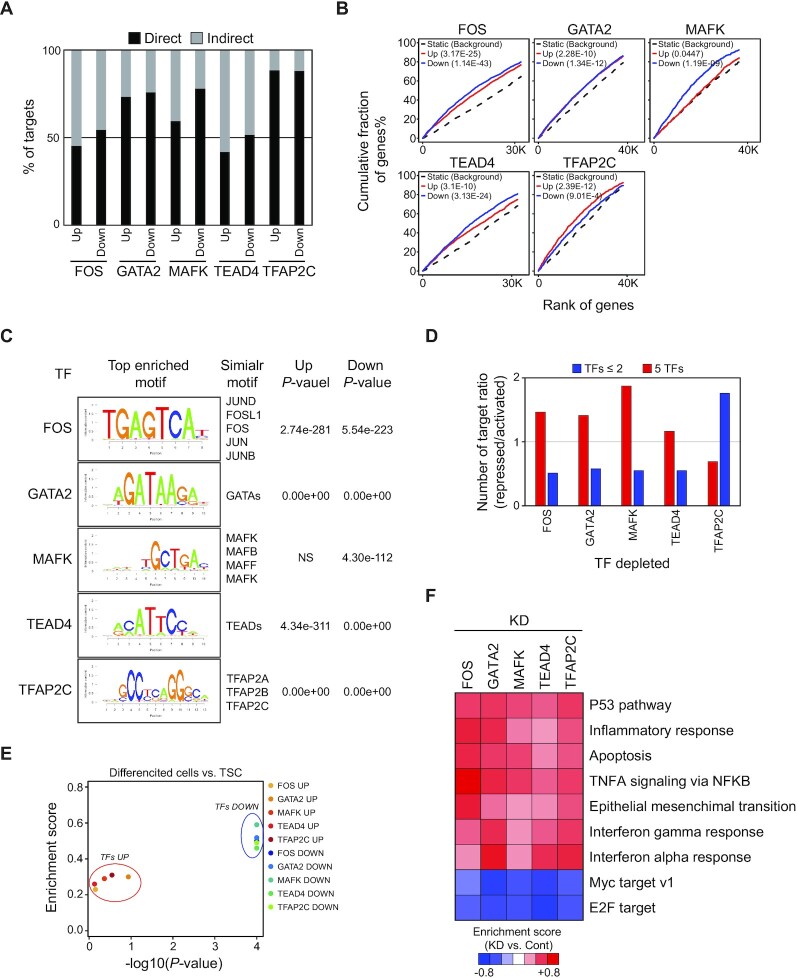
TSC-pivotal TFs directly activate proliferation-associated genes but repress developmental genes. (**A**) Percentage of up- and down-regulated direct and indirect targets of SE-TFs. (**B**) Activation and repression activity of TFs predicted by BETA. Red and blue lines indicate the up- and down-regulated genes upon KD of a TF, respectively. The dashed line presents genes that were not differentially expressed as background. The *P*-value shown in the top left indicates the significance of the Up or Down gene group relative to the Static group as determined by the Kolmogorov–Smirnov test. (**C**) Enriched motifs in the direct targets of individual TFs. (**D**) The ratio of repressed to activated genes upon depletion of a TF. Red and blue indicate genes bound by either five TFs or <2 TFs. The *x*-axis shows the TFs depleted. (**E**) Gene set enrichment analysis (GSEA) of up- and down-regulated genes upon depletion of TFs between human TSCs and differentiated cells. (**F**) A heatmap showing enriched GO terms upon KD of a TF. Enrichment scores were obtained from GSEA (KD versus Control).

To gain insights into the molecular functions of the five TFs in TSCs, we knocked down the TFs with shRNAs. After confirming a >70% reduction in the level of each TF using RT–qPCR (Supplementary Figure S5A), we profiled global transcriptomes and identified several hundred DEGs in each TF-depleted TSC compared with an empty control (Supplementary Figure S5B; Supplementary Table S6). First, we inspected whether the TFs directly activate or repress their target genes by examining the overlap of the targets of the TFs with the DEGs. Overall, >50% of DEGs were direct targets of a TF (Supplementary Figure S5C). We further classified up-regulated and down-regulated DEGs into direct and indirect target groups. As shown in Figure 5A, a similar number of direct targets was observed in up-regulated and down-regulated genes upon KD of the TFs, suggesting that these TFs can function as both activators and repressors. We also confirmed our observation using the Binding and Expression Target Analysis (BETA) program which integrates TF binding data with DEGs to statistically infer direct target genes (46). In agreement with the overlap analysis, all five TFs functionally serve as both activators and repressors. Nevertheless, FOS, MAFK and TEAD4 are prone to activate their target genes, while TFAP2C is slightly skewed to its repressive role (Figure 5B). Motif analysis disclosed that the regulatory loci of a TF’s targets were enriched with a corresponding motif to the TF regardless of up-regulated or down-regulated genes, except for MAFK-up-regulated targets, implying that the TF can directly activate and repress their targets (Figure 5C).

To test whether co-occupancy of TFs affects the activation or repression of their target genes, we compared the gene activity of five TF-bound genes with no more than two TF-bound genes when a TF is depleted. Intriguingly, depletion of either one of these TFs (FOS, MAFK, GATA2 and TEAD4) preferentially down-regulated the five TF-bound genes but up-regulated no more than two TF-bound genes. Conversely, KD of TFAP2C showed an opposite pattern (Figure 5D). These results imply that the TFs can function as either activators or repressors depending on the co-occupancy status of their target genes. To dissect the functional roles of an individual TF, we performed gene set enrichment analysis (GSEA) (47). As shown in Figure 5E, the down-regulated genes upon KD of these TFs (TFs DOWN) showed significantly higher expression in TSCs than in differentiated cells, indicating that these TFs activate the genes in TSCs. Moreover, while the targets of MYC and E2F that can promote cell proliferation were down-regulated in these TF-depleted cells, apoptosis and epithelial–mesenchymal transition that occur during TSC differentiation were induced (Figure 5F). Through flow cytometry analysis of annexin V and caspase 3 activity, we confirmed that the cells upon KD of the five TFs presented increased apoptosis, even though there was some degree of difference, suggesting that the depletion of these TFs induces apoptosis (Supplementary Figure S5D). Collectively, these results suggest that the five TSC-pivotal TFs collaboratively maintain TSCs by directly activating self-renewal genes, while repressing differentiation-related genes.

### Commonality and discrepancy of the roles of TSC-pivotal TFs in human and mouse TSCs

**Figure 6.**
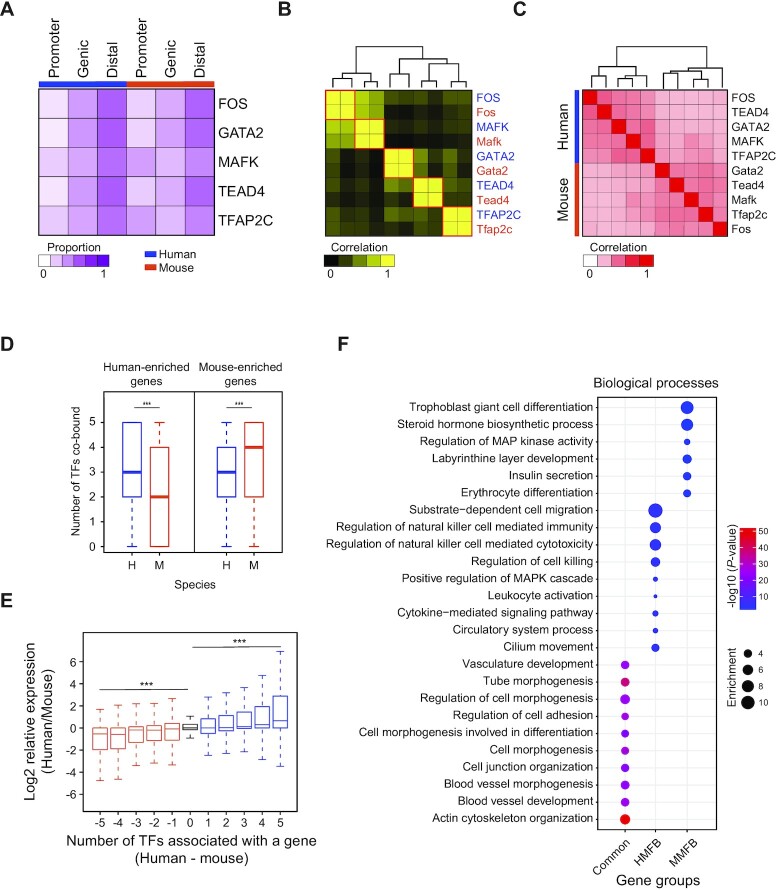
TSC-pivotal TFs regulate common and unique target genes between human and mouse TSCs. (**A**) A heatmap presenting the distribution of TSC-pivotal TF-binding sites across the genome in human and mouse TSCs. (**B**) A heatmap showing the correlation of the occurrence of TF motifs in the target TFs in human and mouse TSCs. Blue and orange colors indicate TFs in humans and mice, respectively. (**C**) A heatmap displaying overlaps of TF target genes in human and mouse TSCs. (**D**) Box plots showing the numbers of TSC-pivotal TFs that are bound on human- and mouse-enriched genes in human and mouse TSCs. H and M indicate humans and mice, respectively. (**E**) Box plots presenting the relative expression of genes that are classified by the difference in the number of TSC-pivotal TFs bound between human and mouse TSCs. (**F**) GO terms of biological processes enriched in common targets of six TFs between human and mouse TSCs and unique targets of each species. MMFB and HMFB indicates mouse-multiple-factors-bound and human-multiple-factors-bound, respectively.

Previously, we identified mouse TSC-pivotal SE-TFs and the targets of a subset of the TFs (16). We identified that the five TFs (FOS, GATA2, MAFK, TEAD4 and TFAP2C) are also key regulators in mouse TSCs. Since human and mouse placentas have common and distinct characteristics in structure and gene expression patterns (17–19), we compared the binding sites and the target genes of the five TFs between human and mouse TSCs to dissect the shared and unique functions of these TFs in each species. These TSC-pivotal TFs prefer to bind distal enhancers regardless of their origins (Figure 6A). The motif of each TF was enriched at the center of a TF-binding site and well conserved in both species (Figure 6B; Supplementary Figure S6A). We also explored the target correlation among the TFs between the species. Although a stronger target correlation was observed within the species, many target genes were also highly overlapped between the species (Figure 6C), indicating common functions of the TFs between human and mouse TSCs.

To investigate to what extent these five TFs influence the species-specific gene expression, first we classified the genes into two groups (human-enriched and mouse-enriched gene groups) with a criterion of 2-fold higher gene expression by comparing the transcriptomes of human TSCs with those of mouse TSCs. Next, we examined the number of TFs associated with a gene. The number of co-bound TFs on genes enriched in humans was significantly higher in human TSCs than in mouse TSCs, whereas mouse TSCs hold a higher number of TFs binding on the genes enriched in mice (Figure 6D). Moreover, we observed a gradual increase in gene expression according to the increment in TFs co-bound in both species (Figure 6E). All these results indicate that TF co-occupancy controls species-specific gene expression. We further dissected target gene groups based on the number of TFs co-bound among the five TFs plus EP300 in both species. We defined the genes regulated by all six factors in both species as common, human-multiple-factors-bound (HMFB) genes as those that are regulated by the six factors in human but bound by no more than two factors in mouse, and mouse-multiple-factors-bound (MMFB) genes as those regulated by the six factors in mouse but bound by no more than two factors in human. Based on these criteria, we defined 1206, 775 and 407 genes belonging to the common, HMFB and MMFB gene groups, respectively (Supplementary Table S7). GO analyses revealed that common genes were significantly enriched for placental development-associated terms, including cell junction organization, cell morphogenesis involved in differentiation, and blood vessel development. While HMFB genes were enriched for the regulation of natural killer cell-mediated immunity, MMFB genes were over-represented in trophoblast giant cell differentiation and labyrinthine layer development (Figure 6F). Collectively, these TSC-pivotal TFs regulate both distinct and shared gene expression programs between mouse and human TSCs.

## DISCUSSION

Developmental processes are regulated by the collaborative action of tissue-specific enhancers and TFs. In this study, we identified tens of thousands of enhancers, and several hundred SEs as well as SE-associated genes across the genome in human TSCs. Among the SE-associated genes, we found multiple previously known trophoblast-pivotal TFs, including MSX2 that was recently characterized as a repressor of ST differentiation (39). Importantly, we identified many uncharacterized SE-TFs which are highly likely to be other important players in human placental development. Pathway analysis disclosed that SE-associated genes are the most significantly enriched for the Hippo signaling pathway that is known to control cell stemness, differentiation and fate decision (48). In line with our observation, recent studies show that regulators of the Hippo pathway (TEAD4 and YAP1) play crucial roles in the self-renewal of trophoblast progenitors and secure the survival of early post-implanted human and mouse embryos (32,49). Interestingly, the pathway in cancer was also significantly enriched among the SE-associated genes, suggesting that the genes involved in the cancer pathway are also active in TSCs but do not induce tumorigenesis. It will be interesting to study how TSCs can circumvent tumorigenesis even though many genes implicated in the cancer pathway are active in TSCs.

In accordance with a previous mouse TSC study (16), SE-associated genes are over-represented for the molecular functions of TF binding. This observation corroborates evidence to support the idea of identifying TSC-pivotal TFs by defining SE-associated TFs in TSCs. We identified 76 putative TSC-pivotal TFs in human TSCs and investigated the global binding sites of five TSC-pivotal SE-TFs that are also regulated by SEs in mouse TSCs (16). Given the fact that TFs can enhance their binding on the genome by cooperatively interacting with DNA, ensuring robust and stable gene expression (50), we reasoned that the five TFs may function cooperatively to form an enhanceosome and act as a single discrete functional module to secure TSC-specific gene expression programs. In agreement with this notion, our analysis showed that the five TFs not only co-occupy their target genes involved in placental development but also regulate one another. It can also be speculated that these TFs act as protein complexes or within compact enhancers because their binding sites are nearly coincident. Of note, PE-deregulated genes are also co-bound by the five TFs, suggesting that co-occupancy of the TFs is one of the key mechanisms to regulate cell type-specific and PE-associated gene expression. Therefore, it will be necessary to investigate combinatory interplays of multiple TSC-pivotal TFs to understand the basis underlying placental development and disorders. It is worth noting that, unlike the other four TFs we investigated, the level of MAFK is higher in STs than in TSCs and CTs (Supplementary Figure S7A). This observation implies that MAFK can play lineage-specific roles, depending on the cellular context.

A loss-of-function study of the five TFs revealed that they could function as both activators and repressors. Nevertheless, depletion of TFAP2C tended to activate multiple TFs-bound genes, unlike others, suggesting that TFAP2C can function as a repressor in a subset of genes. Since multiple TF-bound targets are likely to be activated (43), it is possible that TFAP2C may attenuate or fine-tune the level of TSC-specific genes, instead of completely repressing the genes, as shown in the role of Tgif1 in mouse embryonic stem cells (51). TFAP2C might maintain the proper level of a subset of TSC-specific genes by counterbalancing the gene activity in TSCs.

The mouse and human placenta differ in structure, cell types, endocrine functions and the length of gestation (52). Although our target comparison of TSC-pivotal TFs in human and mouse TSCs showed strongly enriched common functions of TSC-pivotal TFs, there are also distinct roles of the TFs in each species. For example, MMFB genes were enriched for GO terms of trophoblast giant cell differentiation and labyrinthine layer development. In line with this observation, mouse trophoblast giant cells are not nearly as invasive as the human equivalent cell, EVTs, and the mouse labyrinth is different from the STs in humans (53). These functionally conserved and divergent roles of TFs in TSCs may explain the phenotypic variations of the placenta, reflecting the evolutionary differences in the placenta. Although much work is still required to elucidate the functional significance of many TSC-active TFs, our study provides significant insights into how TSC-pivotal TFs modulate trophoblast-active gene expression in human TSCs.

## DATA AVAILABILITY

Raw and processed ChIP-seq and RNA-seq data have been deposited to the public server GEO database under the accession number GSE208539.

## Supplementary Material

gkad215_Supplemental_Files
