# Supplementary material for: Super-enhancer-associated transcription factors collaboratively regulate trophoblast-active gene expression programs in human trophoblast stem cells
Source: Nucleic Acids Res. 2023 Mar 23;51(8):3806–19. doi: 10.1093/nar/gkad215 (PMC10164554; doi:10.1093/nar/gkad215)
Supplement: gkad215_Supplemental_Files [file gkad215_supplemental_files.zip › Supplementary_Data.pdf]

Supplementary Data

Supplementary Table Legend

Supplementary Table S1. Primers for RT-qPCR and shRNA sequences
Supplementary Table S2. Lists of super-enhancers (SEs), SE-associated genes, and SE-associated TFs defined by P300
Supplementary Table S3. Lists of super-enhancers (SEs), SE-associated genes, and SE-associated TFs defined by H3K27ac
Supplementary Table S4. The binding sites of an individual TF across the genome in TSCs.
Supplementary Table S5. Unique and co-bound sites among 5 TFs. 1, 2, 3, 4, and 5 in TF's co-bound. Column indicates FOS, GATA2, TFAP2C, Tead4, and MAFK, respectively.
Supplementary Table S6. Up- and down-regulated genes upon knockdown of a TF
Supplementary Table S7. Mouse-multiple-factors-bound (MMFB), human-multiple-factors-bound (HMFB), and common genes classified by the status of multiple TSC-pivotal TFs' co-binding in human and mouse TSCs.

Supplementary Figures

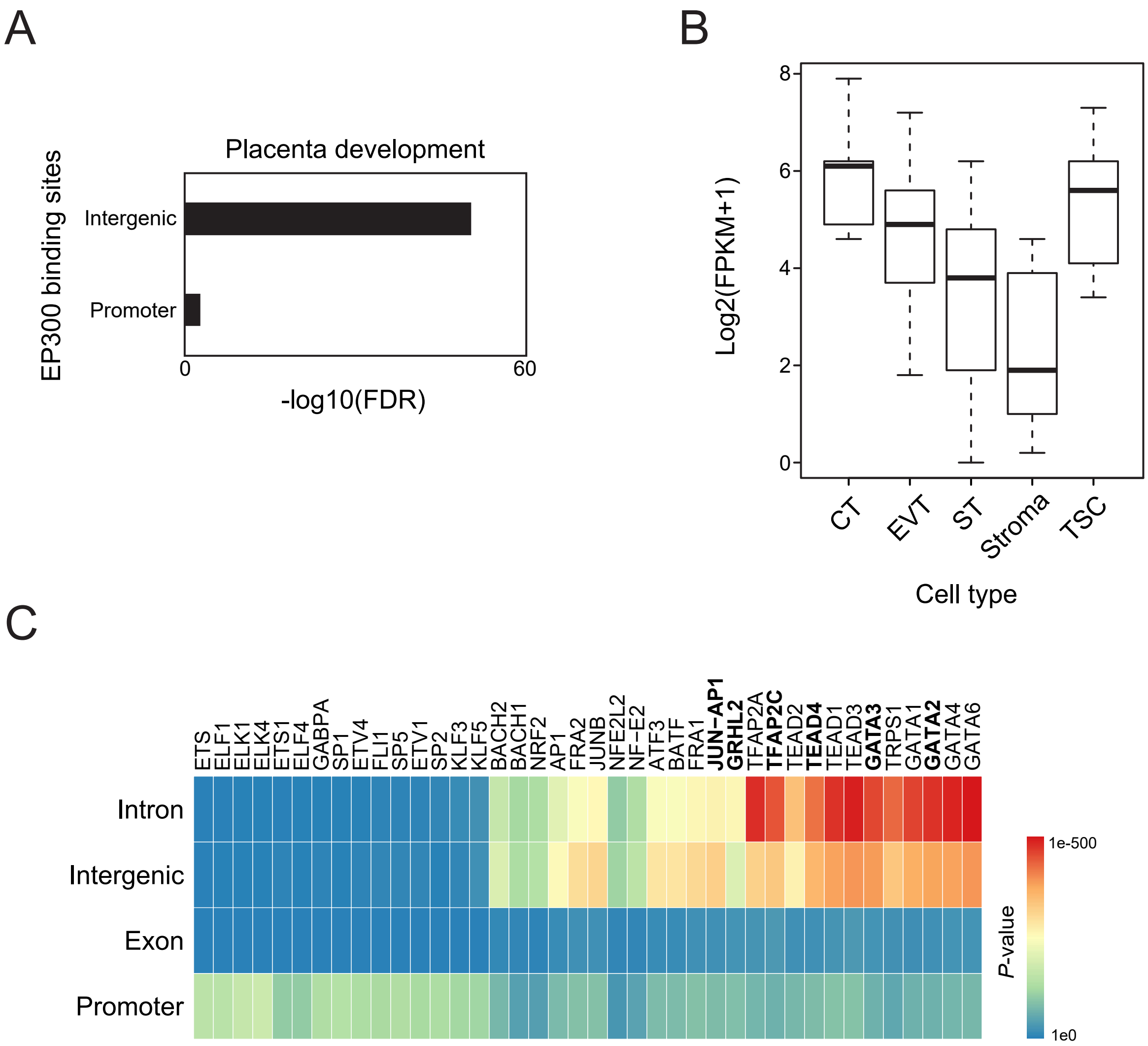

Supplementary Figure S1. ChIP-seq of EP300 in human TSCs identified TSC-specific enhancers. (**A**) A bar graph showing the enriched GO term of placenta development of the EP300 binding sites in promoter and intergenic regions for placenta development. (**B**) A boxplot presenting the expression distribution of top 10 TFs whose motif are enriched in EP300 binding sites in various cell types. Gene expression data were obtained from published data (1). (**C**) A heatmap depicting the enriched motifs of TFs in 4 different genomic regions of EP300 binding sites, including promoter, intron, intergenic, and exon.

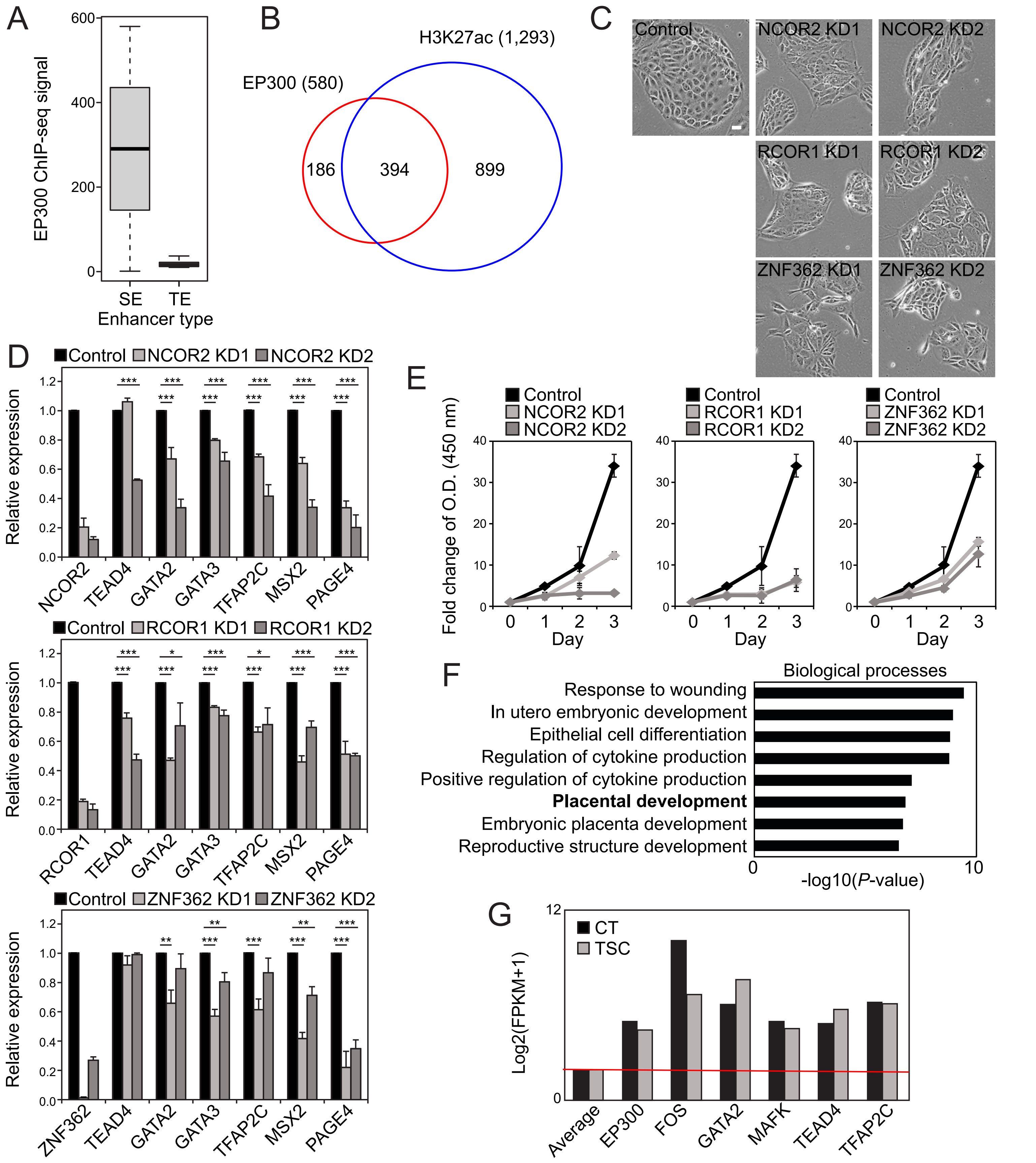

Supplementary Figure S2. Super-enhancers (SEs)-associated genes contribute to human TSC maintenance. (**A**) A Boxplot presenting EP300 ChIP-seq signals in SEs and typical enhancers (TEs). (**B**) A Venn diagram displaying the overlap between SEs defined by EP300 and H3K27ac. (**C**) Morphology of TSCs upon depletion of individual TFs. (**D**) Relative expression of TSC-active genes upon KD of putative TSC-pivotal candidate TFs. The *P*-value was calculated using Student's t-test. *, **, and *** indicate $P < 0.05$, $P < 0.01$, and $P < 0.001$, respectively. (**E**) Cell proliferation rate of TF-KD and control cells. (**F**) A bar graph showing the enriched GO terms of biological processes in SE-associated genes. (**G**) Expression of TFs in CTs and human TSCs. Gene expression data were obtained from published data (1).

A

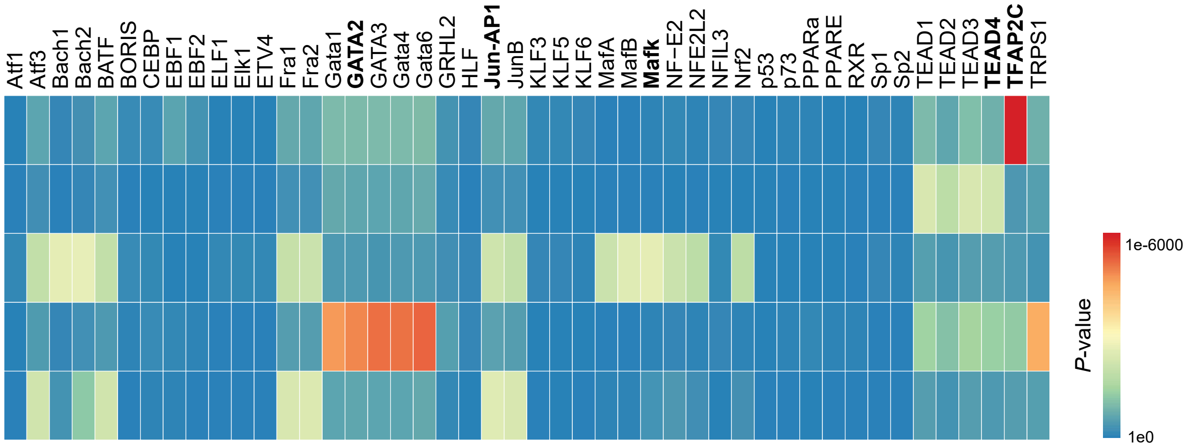

Supplementary Figure S3. TSC-active TFs' motifs were enriched in the binding site of TSC-pivotal TFs. (**A**) A heatmap depicting the enriched motifs (X-axis) of the binding sites of a TF shown in Y-axis.

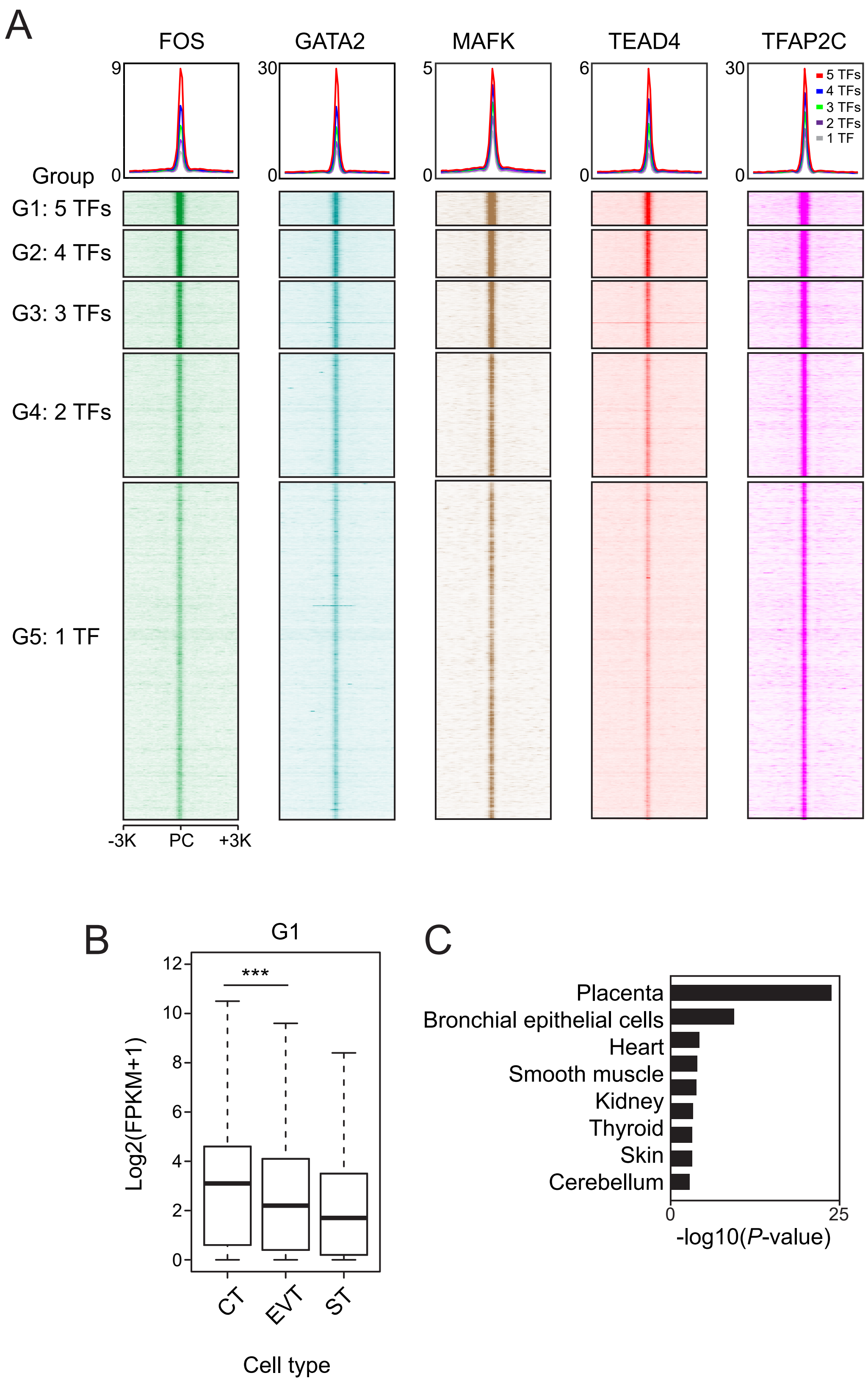

Supplementary Figure S4. TSC-pivotal TFs colloboratively bind to regulate TSC-active genes. (**A**) Heatmaps presenting TFs occupancy signals at the center of peaks belonging to each group that is classified based on the number of TF co-bound. PC indicates a peak center. (**B**) A Boxplot showing the distribution of gene expression of the G1-associated genes in various trophoblast cell types obtained from the placenta. *P*-value was calculated by the Wilcoxon rank sum test. *** indicate $P < 0.0001$. Gene expression data were obtained from published data (1). (**C**) A bar graphs showing cell types enriched with G1-associated genes.

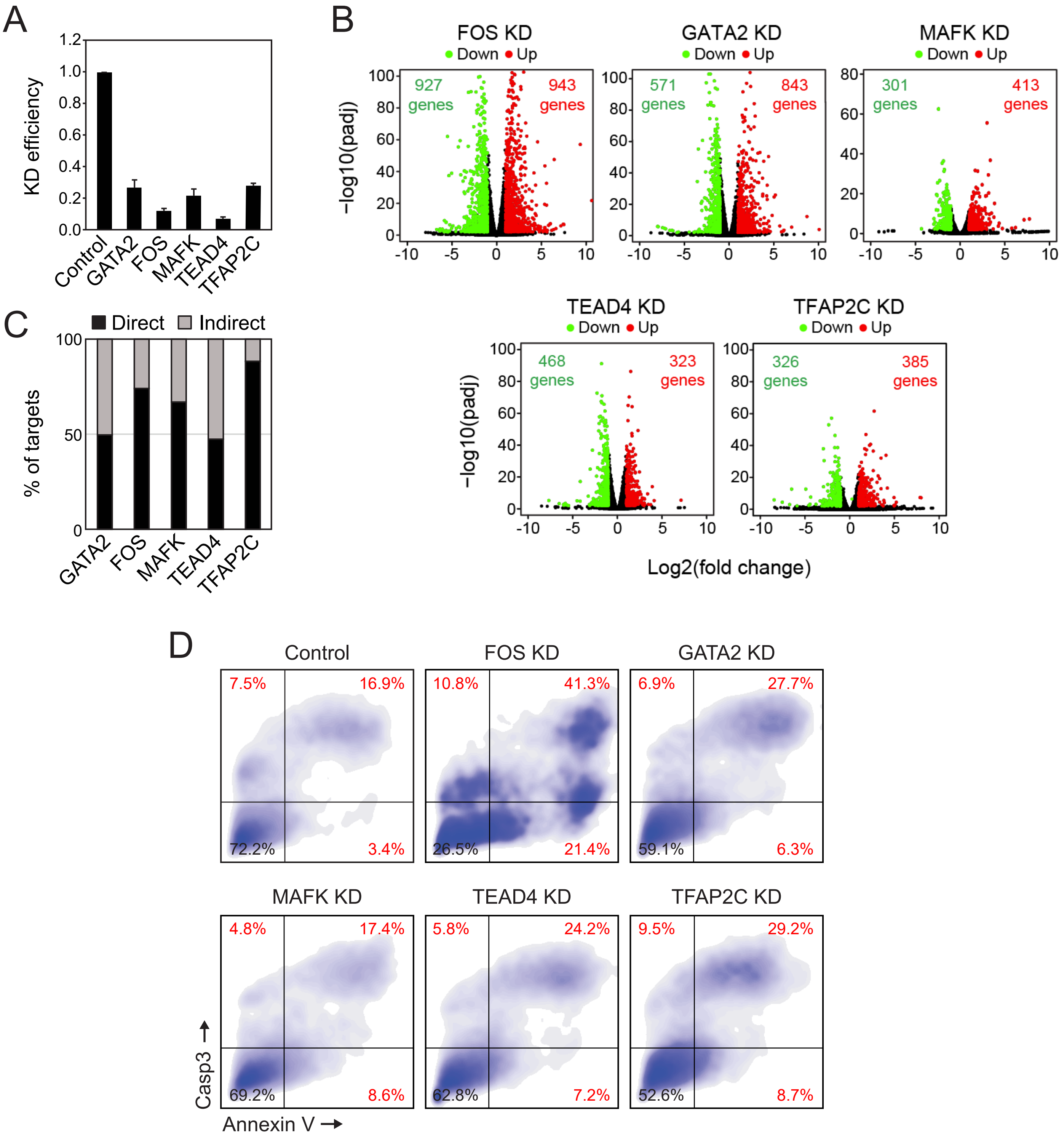

Supplementary Figure S5. TSC-pivotal TFs directly regulate gene expression profile in human TSCs. (**A**) Knockdown efficiency of SE-TFs using shRNAs. Error bars depict a standard error of mean (SEM) with three biological replicates. Control indicates an empty vector. (**B**) Volcano plots illustrating significantly upregulated and downregulated genes upon KD of TFs. Cut-off criteria (Fold change > | 1 | and *P*-value < 0.01) were used. (**C**) Percent of direct and indirect TFs' targets. (**D**) Flow cytometry density plots presenting the cell population having fluorescent signals from annexin V (conjugated to CF594) and caspase 3 activity (NucView 488) upon KD of an individual TF in TSCs.

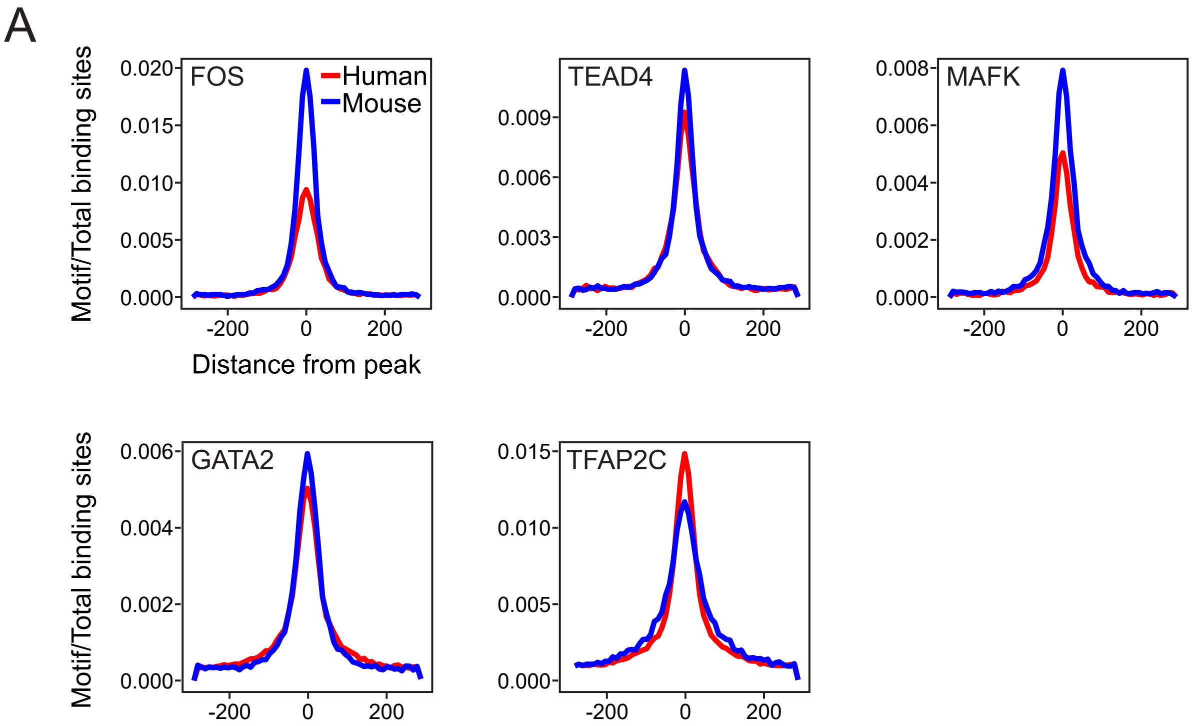

Supplementary Figure S6. Binding of the TSC-pivotal TFs on their motifs. (A) Line graphs showing the distribution of a TF's motif occurrence around the center of a TF's binding sites.

A

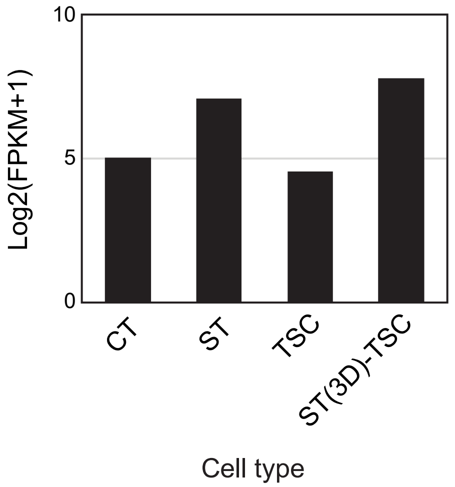

Supplementary Figure S7. MAFK is highly expressed in both CT/TSC and ST. (**A**) Expression of MAFK in different trophoblast lineage. ST(3D)-TSC indicates that ST differentiated from TSCs in a 3D culture. Gene expression data were obtained from published data (1).

References

1. Okae, H., Toh, H., Sato, T., Hiura, H., Takahashi, S., Shirane, K., Kabayama, Y., Suyama, M., Sasaki, H. and Arima, T. (2018) Derivation of Human Trophoblast Stem Cells. Cell Stem Cell, 22, 50-63.e56.
